# Effects of Treg cells and IDO on human epithelial ovarian cancer cells under hypoxic conditions

**DOI:** 10.3892/mmr.2014.2893

**Published:** 2014-11-07

**Authors:** JUN LIU, HAIYAN ZHANG, LUOQI JIA, HONG SUN

**Affiliations:** Department of Gynecology, Obstetrics and Gynecology Hospital, Fudan University, Shanghai 200090, P.R. China

**Keywords:** epithelial ovarian cancer, IDO, regulatory T cells, hypoxia

## Abstract

The aim of the present study was to explore the effect of hypoxia on ovarian cancer. A total of 6 samples were analyzed: SKOV3-IP cells (ovarian cancer cell line); SKOV3-IP and regulatory T (Treg) cells; SKOV3-IP and cytotoxic T lymphocytes (CTLs); SKOV3-IP and natural killer (NK) cells; SKOV3-IP co-cultured with CTLs and Treg cells; and SKOV3-IP co-cultured with Treg cells and NK cells. The expression of indoleamine 2,3-dioxygenase (IDO) was detected by reverse transcription-polymerase chain reaction (RT-PCR) and western blot analysis. An enzyme-linked immunosorbent assay (ELISA) was used to detect the concentration of transforming growth factor-β (TGF-β), interferon-γ (IFN-γ), interleukin-2 (IL-2), interleukin-10 (IL-10), and perforin. Moreover, ovarian cancer cell apoptosis and invasive ability were examined using flow cytometry and a Transwell chamber assay. IDO expression was significantly reduced in hypoxia and enhanced by Treg cells. Treg cells inhibited the IL-2, IFN-γ and perforin secretion, and significantly (P<0.05) increased the IL-10 and TGF-β levels. The effects of Treg cells were enhanced with prolongation of the cell exposure to hypoxic conditions. In addition, Treg cells attenuated the promotive effect of CTLs and NK cells on cancer cell apoptosis. In addition, Treg cells significantly increased the SKOV3-IP invasive ability (P=0.00109) under hypoxic conditions. Our results suggest that IDO and Treg cells may serve as important therapeutic targets for patients with ovarian cancer.

## Introduction

Ovarian cancer is one of the most serious malignant tumors that threaten the health of women, and the 5-year survival rate in advanced ovarian cancer patients is estimated at only at 30.6% (1). Therefore, improving the survival rate of these patients is a major clinical issue. Since the 1990s, research studies have shed light on immunotherapy, which may be the most important tool of the 21st century (2). The regulatory functions of CD4^+^ CD25^+^ regulatory T (Treg) cells in the maintainance of immune homeostasis, tumor immunity, allergic reactions and microbial infection are well established (e.g., 3). Active CD4^+^ CD25^+^ Treg cells can effectively inhibit the functions of natural killer (NK) cells, B cells and dendritic cells (DCs) based on cell-cell contact mechanisms or via the production of soluble factors, such as transforming growth factor-β (TGF-β) and interleukin-10 (IL-10) (4).

The expression of indoleamine 2,3-dioxygenase (IDO) has been shown to be significantly increased in a variety of tumor cells (5). Moreover, a recent study reported that IDO can suppress the immune function of T cells by inducing the differentiation of naive T cells to Treg cells (6). A number of studies have focused on the role of IDO in cancer development and therapy. Mei *et al* (7) reported that IDO1 enhances survival and invasiveness of endometrial stromal cells via the activation of the JNK signaling pathway. Chen *et al* (8) demonstrated that attenuation of immune suppression via inhibition of the IDO1 enzymatic activity may be an important mechanism underlying polyphenol-mediated chemoprevention or combinatorial cancer therapy. In addition, a previous study reported that certain phytochemicals markedly reduce the IDO1 activity, and that this inhibition may at least in part explain their anti-cancer properties (9). Furthermore, Wang *et al* (10) revealed that downregulation of IDO controls ovarian cancer progression by activating NK cells, and proposed that IDO may be potentially useful in the therapy of ovarian cancer. de Jong *et al* (11) found that IDO-induced immune escape may play an important role in ovarian cancer. 1-Methyl-D-tryptophan may promote anti-tumor immune escape by increasing the IDO1 level in cancer cells (12). It is generally believed that the combination of IDO and DCs is the major cause of tumor cell immune tolerance induced by Treg cell proliferation (13). Due to the important roles played by IDO and Treg cells, an important body of research has focused on the identification of factors that may affect their activity, including hypoxia. Hypoxia is considered one of the basic features of the tumor microenvironment in the body (14). In the hypoxic environment, the ovarian cancer cell adhesion ability was shown to be decreased, while invasive ability is increased, inducing peritoneal metastases or recurrence (15). Although a number of studies have been published on hypoxia, the relationship and interaction between the tumor hypoxic microenvironment and tumor immunity still remains unclear.

In this study, the expression of IDO in ovarian cancer cells was inhibited by hypoxia and enhanced by Treg cells. In addition, the expression of interleukin-2 (IL-2), interferon-γ (IFN-γ), perforin, IL-10 and TGF-β was significantly changed in cultures containing Treg cells under hypoxic conditions. Furthermore, our study indicated that Treg cells may significantly enhance ovarian cancer cell apoptosis and invasive ability, especially in hypoxia. Overall, our study explored the different effects of IDO and Treg cells on ovarian cancer cells under hypoxic conditions, and suggests that targeting IDO and Treg cels may constitute a suitable therapeutic route for ovarian cancer.

## Materials and methods

### Cell cultures and study groups

The epithelial ovarian cancer cell line SKOV3-IP was provided the by Institute of Obstetrics and Gynecology Hospital at Fudan University. Treg cells, NK cells and cytotoxic T lymphocytes (CTLs) were derived from peripheral blood of healthy adult females.

SKOV3-IP cells (10^6^/ml) were inoculated with Dulbecco’s modified Eagle’s medium with Nutrient Mixture F-12 (DMEM-F12) supplemented with 10% Gibco^®^ fetal bovine serum (FBS) and Gibco^®^ 1% penicillin/streptomycin (all from Thermo Fisher Scientific, Waltham, MA, USA), and cultured at 37°C, in a 5% CO_2_ incubator. The medium was replaced every other day. After cells had reached 80–90% confluence, they were digested by a 0.25% trypsin-ethylene diamine tetraacetic acid solution (Gibco^®^, Thermo Fisher Scientific) and transferred to a new flask. Aerobically cultured cells were placed in a 37°C incubator (95% air, 5% CO_2_). Hypoxia-cultured cells were sealed in an anaerobic culture tank (1% O_2_, 5% CO_2_ and 94% N_2_ ) at 37°C.

The cells were divided into 6 groups: SKOV3-IP; SKOV3-IP and Treg cells; SKOV3-IP and CTLs; SKOV3-IP and NK cells; SKOV3-IP co-cultured with CTL and Treg cells; and SKOV3-IP co-cultured with NK and Treg cells.

### Reverse transcription- polymerase chain reaction (RT-PCR)

Total RNA was extracted using the Invitrogen™ TRIzol reagent (Thermo Fisher Scientific) following the manufacturer’s instructions, and the quantity of RNA was analyzed by UV spectrophotometry. RNA (4 μg) was reverse transcribed to cDNA with Moloney murine leukemia virus reverse transcriptase in a 30-μl reaction volume using oligo (dT)18 primers, RNase inhibitor and buffers from the All-in-oneTM First-strand cDNA synthesis kit (GeneCopoeia, Rockville, MD, USA), following the manufacturer’s instructions. The synthesized cDNA was used for PCR, conducted on a DNA thermocycler (Takara Bio, Inc., Shiga, Japan) with the following conditions: Initial denaturation at 95°C for 3 min, and then 35 cycles amplication (95°C for 20 sec, 60°C for 30 sec and 72°C for 30 sec. The reaction was performed on a 25-μl volume, containing Taq DNA polymerase and PCR buffer provided in the Phusion Blood Direct PCR kit (Finnzymes, Espoo, Finland), a dNTP mix and primers for each gene. The glyceraldehyde-3-phosphate dehydrogenase gene (*GAPDH*) was used as the internal control. Primer sequences are listed in Table I. Quantitative PCR was conducted on an iQ5 Multicolor Real-Time PCR Detection System (Bio-Rad Laboratories, Hercules, CA, USA) using a SYBR Green Real-time PCR Master Mix (Takara Bio, Inc.). Data were calculated using the 2^−ΔΔCt^ method normalized to the individual internal control level.

### Western blot analysis

The treated cells were washed twice with cold Gibco^®^ phosphate-buffered saline (Thermo Fisher Scientific) and were lysed in RIPA buffer (Biocolor BioScience & Technology Co., Ltd., Shanghai, China) in the presence of a proteinase inhibitor (Kangchen Bio-tech, Shanghai, China). Protein quantification was performed with the BCA-100 protein assay kit (Biocolor BioScience & Technology Co., Ltd.). Samples were subjected to 8% sodium dodecyl sulphate polyacrylamide gel electrophoresis, transferred to polyvinylidene fluoride (PVDF) membranes (EMD Millipore, Billerica, MA, USA) and blocked with 5% fat-free milk for 2 h at room temperature. Equal loading in each blot was confirmed by Coomassie staining (Beyotime Biotechnology, Shanghai, China) of the membrane. The membrane was incubated overnight with a mouse anti-human IDO monoclonal antibody (1:500; Abcam, Cambridge, MA, USA) and mouse anti-human GAPDH monoclonal antibody (1:5,000; Cell Signaling Technology, Inc., Beverly, MA, USA) at 4°C. Then, the membrane was incubated with the horseradish peroxidase (HRP)-labeled secondary antibody (1:7,000) for 1 h at room temperature. Signals were visualized with an enhanced chemiluminescence kit (Amersham Pharmacia Biotech, Piscataway, NJ, USA) and quantified using an Odyssey^®^ imaging system (LI-COR Inc., Lincoln, NE, USA). The protein level of IDO was normalized to the level of GAPDH.

### Enzyme-linked immunosorbent assay (ELISA)

TGF-β, IFN-γ, IL-2, IL-10 and perforin levels were detected in the supernatant of cell culture medium by ELISA using corresponding kits (eBioscience, San Diego, CA, USA) according to the manufacturer’s instructions. The absorbance of the samples was measured on an ELISA plate reader (eBioscience) at a 450 nm wavelength, with a 610–630 nm reference filter. The concentration was determined by standard curve analysis, based on the absorbance of respective standards.

### Apoptotic assay of ovarian cancer cells

To analyze the apoptosis of SKOV3-IP cells, we used the Invitrogen™ Apoptosis Assay kit (Thermo Fisher Scientific) according to the manufacturer’s instructions. Briefly, ~1×10^5^ SKOV3-IP cells were cultured in a 12-well plate. In parallel, co-cultures with 5×10^5^ Treg cells, 1×10^6^ NK cells and 1×10^6^ CTLs per well were established. There were three replicates for each group. The cells in each group were then subjected to different conditions i.e., 72-h aerobic growth, 48-h normoxia and 24-h hypoxia, 24-h aerobic and 48-h anaerobic growth, and 72-h hypoxia. Next, the supernatant and the suspended cells were discarded and the cells were dissociated with trypsin to obtain a single-cell suspension. Flow cytometry (BD Biosciences, Franklin Lakes, NJ, USA) was used to detect the apoptotic rate of ovarian cancer cells under the different culture conditions.

### Invasive ability of ovarian cancer cells

The migratory ability of ovarian cancer cells was assessed using Transwell chambers and Costar^®^ cell culture plates (all from Corning, Tewksbury, MA, USA). The transwell chambers were placed in a 24-well plate. The bottom of the chambers was coated with Matrigel (BD Biosciences), 2×10^5^ SKOV3-IP cells per well were plated in the upper chambers of the 24-well Transwell chamber and then 600 μl DMEM-F12 supplemented with 10% FBS were added to the lower chambers. Following incubation for 24 h at 37°C in 5% CO_2_, cells located on the upper membranes were removed with cotton swabs. The cells that had invaded the lower surface of the membrane were fixed in ethanol and stained with crystal violet. Images of invading cells were acquired under a Leica DC 300F microscope (Olympus, Tokyo, Japan) in five random fields (magnification, ×100). The invasive ability of the cells in each group was determined by the average number of invading cells in the five fields.

### Statistical analysis

All experiments were repeated at least three times and each experiment was performed at least in duplicate. The results were presented as mean ± standard deviation (SD). Statistical analysis was performed using a one-way analysis of variance (ANOVA) and χ^2^ tests, implemented in the SPSS 11.5 (SPSS Inc., Chicago, IL, USA) or Excel (Microsoft, Bellevue, WA, USA) software. P<0.05 was considered to indicate statistically significant differences.

## Results

### Expression of the IDO mRNA under different conditions

**Figure 1:**
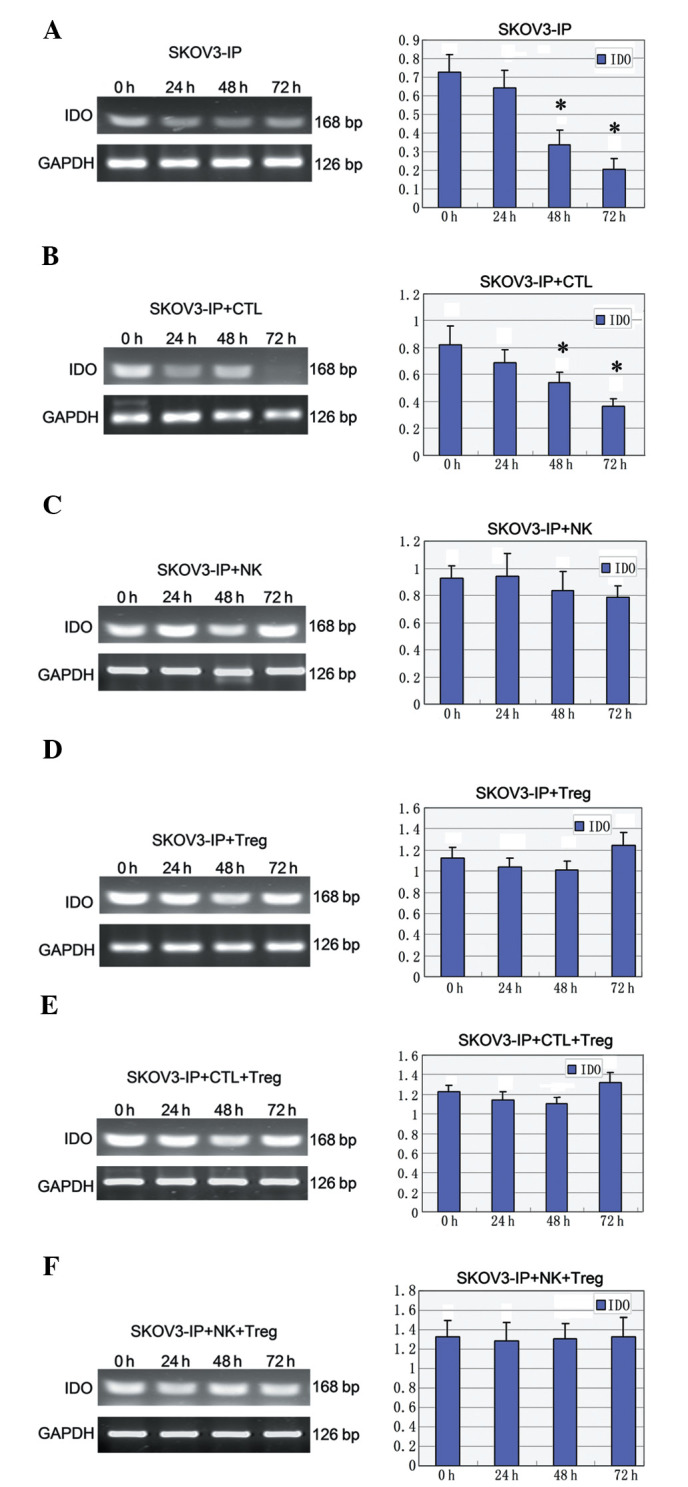
Expression of the *IDO* mRNA under different conditions. *IDO* levels in the (A) SKOV3-IP, (B) SKOV3-IP + CTL, (C) SKOV3-IP + NK, (D) SKOV3-IP + Treg, (E) SKOV3-IP + CTL + Treg, and (F) SKOV3-IP + NK + Treg groups. ^*^P<0.05 vs. 0 h; IDO, indoleamine 2,3-dioxygenase; CTL, cytotoxic T lymphocytes; NK, natural killer cells; Treg, regulatory T cells; GAPDH, glyceraldehyde-3-phosphate dehydrogenase.

The *IDO* mRNA expression level showed a decreasing trend (P<0.05) with prolonged exposure to hypoxia in SKOV3-IP (Fig. 1A), and in SKOV3-IP + CTL cells (P<0.05) (Fig. 1B). When SKOV3-IP cells were co-cultured with NK cells, the *IDO* mRNA expression level was slightly increased in the 24-h hypoxia group and was again decreased at 48 and 72 h (Fig. 1C). In the co-cultured system of SKOV3-IP and Treg cells, the level was decreased in the early hypoxia and increased at 72 h of hypoxia (Fig. 1D); similar changes were observed in the SKOV3-IP + CTL + Treg group (Fig. 1E). In addition, a slight fluctuation in the *IDO* level was observed in the SKOV3-IP + NK + Treg group (Fig. 1F).

### Expression of the IDO protein

**Figure 2:**
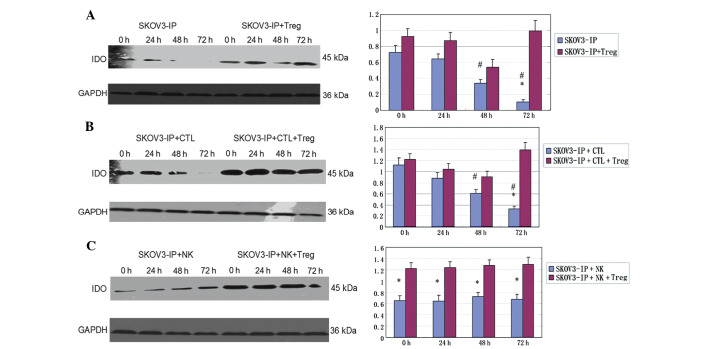
Expression of the IDO protein in SKOV3-IP cells under different conditions. (A) SKOV3-IP and SKOV3-IP + Treg, (B) SKOV3-IP + CTL and SKOV3-IP + CTL + Treg, and (C) SKOV3-IP + NK and SKOV3-IP + NK + Treg. ^*^P<0.05 vs. group with Treg cells; ^#^P<0.05 vs. 0 h; IDO, indoleamine 2,3-dioxygenase; Treg, regulatory T cells; CTL, cytotoxic T lymphocytes; NK, natural killer cells.

The expression of the IDO protein was significantly decreased in SKOV3-IP cells along the time of exposure to hypoxia (P<0.05). The IDO level was higher in the SKOV3-IP + Treg group compared to the group of SKOV3-IP cells, and decreased within 48 h, then increased again at 72 h (P<0.05) (Fig. 2A). It is notable that a similar profile was observed in the SKOV3-IP group co-cultured with CTLs and the SKOV3-IP + CTL + Treg group (Fig. 2B). When SKOV3-IP cells were cultured with NK cells, the expression level of IDO slightly changed with the extension of exposure to hypoxia (P>0.05). Addition of Treg cells markedly enhanced the expression of IDO protein (P<0.05), but this effect only slightly changed with time (P>0.05) (Fig. 2C).

### The effect of Treg on ovarian cancer cells

In order to investigate the effect of Treg cells on the immunity of ovarian cancer cells, we measured the concentrations of TGF-β, IFN-γ, IL-2, IL-10 and perforin in the cell supernatants using ELISA.

**Figure 3:**
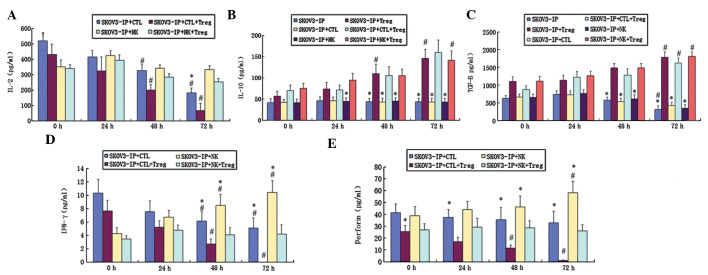
The effect of Treg on the expression of different proteins in ovarian cancer cells. (A, D and E) Expression of IL-2, IFN-γ and perforin in the SKOV3-IP + CTL and SKOV3-IP + NK groups, respectively, with or without Treg cells. (B and C) Concentration of IL-10 and TGF-β in the SKOV3-IP, SKOV3-IP + CTL and SKOV3-IP + NK groups, respectively with or without Treg cells. ^*^P<0.05 vs. group with Treg cells; ^#^P<0.05 vs. 0 h; Treg, regulatory T cells; IL-2, interleukin-2; IFN-γ, interferon-γ; CTL, cytotoxic T lymphocytes; NK, natural killer cells; IL-10, interleukin-10; TGF-β, transforming growth factor-β.

The concentration of IL-2 in the SKOV3-IP + CTL group with or without Treg cells was decreased with the extension of exposure to hypoxia, and this decrease was significant when Treg cells were present (P<0.05) (Fig. 3A). In the co-cultured systems of SKOV3-IP + NK cells, the concentration of IL-2 fluctuated along the time of exposure to hypoxia, with no statistically significant changes observed (P>0.05) (Fig. 3A). In addition, the secretion of IL-10 in SKOV3-IP cells alone, or co-cultured with CTL or NK cells did not change during the exposure to hypoxia (P>0.05) (Fig. 3B). By contrast, the IL-10 level was found significantly and time-dependently increased when Treg cells were present (P<0.05) (Fig. 3B).

The concentration of TGF-β first increased and then decreased at 24 and 72 h, respectively; this trend was observed in SKOV3-IP, SKOV3-IP + CTL and SKOV3-IP + NK cells (Fig. 3C). When Treg cells were present, the TGF-β level increased with the extension of hypoxia (P<0.05) (Fig. 3C). In addition, the IFN-γ level was decreased in the SKOV3-IP + CTL group and this decrease was enhanced when Treg cells was present (P<0.05) (Fig. 3D). NK cells enhanced the secretion of IFN-γ (P<0.05), and Treg cells inhibited the secretion of IFN-γ; this inhibition become more apparent in the hypoxic state (P<0.05) (Fig. 3D). The expression profile of perforin was similar to that of IFN-γ (Fig. 3E).

### Apoptosis of ovarian cancer cells under different conditions

**Figure 4:**
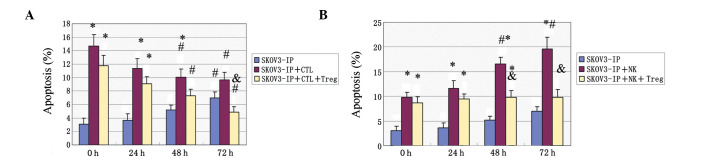
Apoptosis in ovarian cancer cells. (A) The apoptosis percentage in the SKOV3-IP, SKOV3-IP + CTL and SKOV3-IP + CTL + Treg groups and in (B) the SKOV3-IP, SKOV3-IP + NK and SKOV3-IP + NK + Treg groups. ^*^P<0.05 vs. the SKOV3-IP group; ^#^P<0.05 vs. 0 h; ^&^P<0.05 vs. group without Treg cells; CTL, cytotoxic T lymphocytes; Treg, regulatory T cells; NK, natural killer cells.

The apoptotic rate of SKOV3-IP cells was increased with the extension of exposure to hypoxia (P<0.05). In addition, CTLs were found to significantly enhance apoptosis under normoxic conditions (P<0.05). This effect was attenuated with prolonged hypoxia (P<0.05) and was also significant when Treg cells were present (P<0.05) (Fig. 4A). NK cells enhanced SKOV3-IP apoptosis, and this effect was further enhanced with the hypoxic time extension (P<0.05) (Fig. 4B). When Treg cells were added in the culture, the effect was reduced (P<0.05), but was only slightly changed at different time-points (Fig. 4B).

### Invasive ability of ovarian cancer cell lines under normoxic and hypoxic conditions

The number of invading cells was increased when SKOV3-IP cells were co-cultured with Treg cells under normoxic or hypoxic conditions. In addition, hypoxia significantly enhanced the invasive ability of SKOV3-IP cells co-cultured with Treg cells (P=0.00109) or cultured alone (P=0.003171) (Table II).

## Discussion

The expression of IDO in ovarian cancer cells showed a significant trend to decrease at the mRNA and protein level along the time of exposure to hypoxic conditions (Figs. 1 and 2), and these findings are consistent with a previous study (16). The effect of hypoxia on ovarian cancer cell apoptosis and invasive ability was further investigated. The apoptotic rate of ovarian cancer cells was significantly increased under hypoxic conditions (Fig. 4). Previous studies have shown that hypoxia inhibits DNA synthesis, induces cell cycle arrest at the G0-G1 phase, as well as expression changes in cell-cycle proteins. These changes included an increase in p27 expression, a decrease in Rb expression, reduction in the levels of cyclin D1 and E leading to cell-cycle arrest, and inhibition of ovarian cancer cell proliferation. It was further shown that these changes were reverted under normoxia (17,18).

Hypoxia was shown to induce endothelial cell apoptosis through nuclear factor-κB, and to mediate Bcl-2 suppression *in vivo* (19). In our study, hypoxia was found to increase the invasive ability of ovarian cancer cells (Table II). Tumor invasion and metastasis are indicators of the degree of tumor malignancy. The hypoxic microenvironment may induce the expression of genes related to tumor cell invasion, while also reducing cell adhesion and increasing cell motility and invasiveness to promote tumor metastasis (20).

When Treg cells were added to the cultures, the expression level of IDO was increased at both the mRNA and protein level (Figs. 1 and 2). There is some debate on whether Treg cells can promote the expression of IDO, and our findings support the theory that Treg cells enhance IDO expression. In addition, our results showed that IDO expression is reduced by addition of Treg cells during early hypoxia (24–48 h) and is significantly increased at 72 h of hypoxia. This result suggests that the direct effect of hypoxia is to inhibit IDO expression, and that lowly expressed IDO may stimulate immune cells to produce cytokines. Our results are consistent with the findings of Munn and Mellor (21).

To study the effect of Treg cells on the immunity of ovarian cancer cells, we quantified the secretion of the cytokines TGF-β, IFN-γ, IL-2, IL-10 and perforin in the different co-culture groups (Fig. 3). IL-2 plays a crucial role in the maintenance of natural immunologic self-tolerance (22). IL-10, a cytokine with anti-inflammatory properties, has an important role in infection by inhibiting the immune response to pathogens (23). TGF-β has been found to function in vascular development and vascular homeostasis maintenance (24). IFN-γ was reported to play a crucial role in autoimmunity (25). Unlike relatively redundant individual granzymes, functional perforin is essential to cytotoxic lymphocyte function and immune regulation in the host (26). In our study, the levels of IFN-γ, IL-2, IL-10 and perforin were decreased when Treg cells were present in the culture. This finding indicates that Treg cells induce changes in the expression of cytokines; this is likely an immune-escape mechanism. Importantly, Treg cells can suppress immune responses and play an important role in the dominant immune escape process in early tumor progression (27).

In addition, the apoptosis of SKOV3-IP cells was studied under different conditions (Fig. 4). Apoptosis plays a crucial role in the pathogenesis of a variety of cardiovascular diseases that are caused by the loss of terminally differentiated cardiac myocytes (28). In addition, in the absence of adequate vasculature, tumor cells undergo hypoxia and starvation, followed by apoptosis. A previous study reported that hypoxia promotes tolerance and angiogenesis via recruiting Treg cells (29). In our study, the promoting effect of CTLs on apoptosis was inhibited by hypoxia, while the effect of NK cells was enhanced under hypoxic conditions. This may be due to changes in the activity of these cells in the hypoxic environment. Moreover, Treg cells significantly inhibited the cytotoxicity of CTLs, an effect that was more obvious under hypoxic conditions. Treg cells also inhibited the effect of NK cells, but this effect slightly changed between normoxia and hypoxia. Our findings on the effects of Treg cells on cancer cell apoptosis are consistent with a previous study, which showed that Treg cells inhibit the function of NK cells, B cells and other immunocytes (30). We propose that these effects are caused by the increased activity of Treg cells in the hypoxic state.

Intraperitoneal dissemination and distant metastasis constitute important complications in ovarian cancer treatment, and are closely related to the invasion of malignant cells (31). Despite the advances in chemotherapy and considerable efforts made to improve early detection, metastasis remains a major challenge in the clinical management of ovarian cancer (31). Hypoxia was demonstrated to reduce ovarian cancer cell adhesion, and promote cancer cell invasion and metastasis (32). Our experiments confirmed that Treg cells increase the number of invading cells by enhancing the invasive ability of ovarian cancer cells under normoxia or hypoxia. However, this enhancing effect was stronger in hypoxia compared to normoxia. In addition, the invasive ability of SKOV3-IP cells was significantly higher in hypoxic compared to normoxic conditions, independently of the presence of Treg cells. Moreover, the SKOV3-IP invasive ability was more enhanced when co-culturing with Treg cells in hypoxia than in any other condition. Our results suggest that Treg cells and hypoxia may induce the immune escape and ovarian cancer cell metastasis, as previously proposed in other studies (33,34).

In summary, in the ovarian cancer microenvironment, IDO and Treg cells may mutually enhance their levels and synergistically act to attenuate the cytotoxic effect of CTLs and NK cells. These events are enhanced when cells are cultured under hypoxic conditions, which indicates that oxygen depletion plays a key role in the immune tolerance and escape. Our findings are helpful for improving the effects of cancer immunotherapy via the amelioration of the hypoxic microenvironment of malignant tumors. However, further investigation is needed to study the effect of hypoxia on immune escape *in vivo*.

## Figures and Tables

**Table I:** Primers used in the present study.

Primer	Forward	Reverse
IDO	5′-TTTGCTAAAGGCGCTGTTGG-3′	5′-CCTTCATACACCAGACCGTCTGA-3′
GAPDH	5′-CGGAGTCAACGGATTTGGTCGATA-3	5′-AGCCTTCTCCATGGTTGGTGAACAC-3′

IDO, indoleamine 2,3-dioxygenase; GAPDH, glyceraldehyde-3-phosphate dehydrogenase.

**Table II:** Effect of hypoxia and Treg cells on the invasive ability of ovarian cancer cells.

No. of migrating cells	SKOV3-IP	SKOV3-IP + Treg
Normoxia	16.77±5.84	38.77±11.26
Hypoxia	33.66±9.73	89.47±22.45
P-value	0.003171	0.001090

Treg, regulatory T cells.

## Abbreviations

NK: natural killer
DCs: dendritic cells
TGF-β: transforming growth factor-β
IL-10: interleukin-10
IDO: indoleamine 2,3-dioxygenase
CTL: cytotoxic T lymphocytes
RT-PCR: reverse transcription-polymerase chain reaction
PVDF: polyvinylidene fluoride
HRP: horseradish peroxidase
ELISA: enzyme-linked immunosorbent assay
SD: standard deviation
IFN-γ: interferon-γ
